# Functional genomic analysis reveals overlapping and distinct features of chronologically long-lived yeast populations

**DOI:** 10.18632/aging.100729

**Published:** 2015-03-07

**Authors:** Margaret B. Wierman, Mirela Matecic, Veena Valsakumar, Mingguang Li, Daniel L. Smith, Stefan Bekiranov, Jeffrey S. Smith

**Affiliations:** ^1^ Department of Biochemistry and Molecular Genetics, University of Virginia School of Medicine, Charlottesville, VA 22908, USA; ^2^ Department of Nutrition Sciences, University of Alabama at Birmingham, Birmingham, AL 5233, USA; ^3^ Nutrition Obesity Research Center, University of Alabama at Birmingham, Birmingham, AL 5233, USA; ^4^ Comprehensive Center for Healthy Aging, University of Alabama at Birmingham, Birmingham, AL 5233, USA

**Keywords:** yeast, chronological aging, stationary phase, gene expression, biomarkers

## Abstract

Yeast chronological lifespan (CLS) is extended by multiple genetic and environmental manipulations, including caloric restriction (CR). Understanding the common changes in molecular pathways induced by such manipulations could potentially reveal conserved longevity mechanisms. We therefore performed gene expression profiling on several long-lived yeast populations, including an *ade4∆* mutant defective in *de novo* purine (AMP) biosynthesis, and a calorie restricted WT strain. CLS was also extended by isonicotinamide (INAM) or expired media derived from CR cultures. Comparisons between these diverse long-lived conditions revealed a common set of differentially regulated genes, several of which were potential longevity biomarkers. There was also enrichment for genes that function in CLS regulation, including a long-lived adenosine kinase mutant (*ado1∆*) that links CLS regulation to the methyl cycle and AMP. Genes co-regulated between the CR and *ade4∆* conditions were dominated by GO terms related to metabolism of alternative carbon sources, consistent with chronological longevity requiring efficient acetate/acetic acid utilization. Alternatively, treating cells with isonicotinamide (INAM) or the expired CR media resulted in GO terms predominantly related to cell wall remodeling, consistent with improved stress resistance and protection against external insults like acetic acid. Acetic acid therefore has both beneficial and detrimental effects on CLS.

## INTRODUCTION

Aging is a multifaceted process caused by breakdowns in many different cellular processes. Damage caused by increased ROS accumulation, defects in mitochondrial function, and decreased genomic stability seem to be common pro-aging stressors [1-3]. Many pro- or anti-aging cellular responses to these stressors are linked to a core set of conserved nutrient sensing and stress response pathways. In mammalian cells, the efficacy of most anti-aging dietary regimes or drug treatments (for example calorie restriction) can be traced back to some component of insulin/IGF, PKA/Ras, AMPK, or TOR signaling [4]. These nutrient signaling pathways mobilize a set of overlapping anti-aging stress responses within the cell that are mediated specifically by the FOXO transcription factors [4, 5]. Similarly, in single cell eukaryotes, specifically *Saccharomyces cerevisiae*, PKA/Ras and TOR signaling also play large roles in longevity control, partially by moderating cellular stress responses through transcription factors like Msn2/4 and Gis1 [4, 6].

The conservation of these core longevity pathways makes *S. cerevisiae* an excellent model organism for studying the effects of individual genetic, chemical, or environmental factors on longevity. Yeast lifespan can be measured either as the number of times a mother cell divides before senescing (replicative lifespan) or as the number of days that cells remain viable in a post-mitotic stationary phase state where they have exited the cell cycle (chronological lifespan). Both have proven to be useful models for determining the potential aging effects of varying genetic and nutritional factors, which are often conserved in higher eukaryotes [6].

Our lab and others have used the yeast chronological lifespan (CLS) system to screen for genetic factors with functional relevance to longevity [7-10]. Many genes were found to be involved in regulating CLS, but there were surprisingly few genes that overlapped between the various screens, indicating that differences in media, growth conditions, or strain backgrounds likely have large effects on cell survival. An example of a growth condition affecting CLS is the finding that extracellular acetic acid accumulates in aging stationary phase yeast cultures and causes poor cell survival [11]. Interestingly, simply buffering the media pH can suppress this negative effect, as does caloric restriction and inhibition of Sch9 signaling [11, 12]. Similar negative effects of media acidification are also observed on chronological senescence of mammalian cell cultures due to lactate accumulation, which can be attenuated by TOR inhibition [13-15], and raising the possibility of conserved aging associated pathways that are triggered by this type of stress. Alternatively, inhibition of TOR or Sch9 signaling in yeast was shown to extend CLS by promoting catabolism of acetic acid, rather than protection against toxicity [16]. Previous results from our lab showed that gene deletions in the *de novo* purine biosynthesis pathway extend CLS, and similar to CR, at least partially through effects on extracellular acetic acid [9], though we could not clearly distinguish between toxicity vs. catabolism mechanisms. Importantly, passaging yeast cells through acidic/high acetic acid conditions during chronological aging also negatively impacts subsequent replicative lifespan [17, 18], implying there is more similarity in their mechanisms than originally believed.

Despite the large variation between various CLS studies, we hypothesized there could still be universal characteristics shared by different populations of chronologically long-lived yeast cells. For example, we previously observed similarities between CR and mutations in the *de novo* purine biosynthesis pathway, such that the CLS of an *ade4∆* mutant was similar to CLS of a WT strain that was calorie restricted [9]. Additionally, CR did not further extend CLS of the *ade4∆* mutant, suggesting they could share certain mechanisms of lifespan extension. To begin addressing this question of overlap, we decided to take a gene expression biomarker approach to identify additional genes and pathways that are relevant to multiple CLS extending conditions. Gene expression profiling data was obtained from four diverse lifespan extending conditions, including (1) CR, (2) treatment with isonicotinamide (INAM), (3) treatment with a concentrate of expired CR media, and (4) the *ade4∆* mutant from our previous analysis of *de novo* purine biosynthesis [9]. After comparing to a normal WT control strain, we identified differential expression of *CWP2*, *ADD66*, *LEU3*, and *FYV6* during stationary phase as general chronological longevity biomarkers across all tested long-lived conditions. Most of the other genes commonly regulated in the microarrays were also found to significantly impact chronological lifespan when deleted, including *ADO1* (adenosine kinase), which links the methyl cycle with *de novo* purine biosynthesis. GO term analysis of the expression data also revealed two distinct mechanisms of CLS extension related to acetic acid and other organic acids. The first, shared by CR and the *ade4∆* mutant, involved rapid consumption of acetic acid as an alternative carbon source during the diauxic shift. The second mechanism, shared by INAM and the CR concentrate, involved cell wall remodeling resulting in improved resistance to acetic acid that accumulates in the growth medium. Overall, the functional data supports both beneficial and negative effects of acetic acid on yeast chronological aging. Additionally, the biomarkers have potential use for future large-scale genetic screens and analysis of small molecule libraries.

## RESULTS

### Shared gene expression changes induced by CR and an *ade4∆* mutation

**Figure 1:**
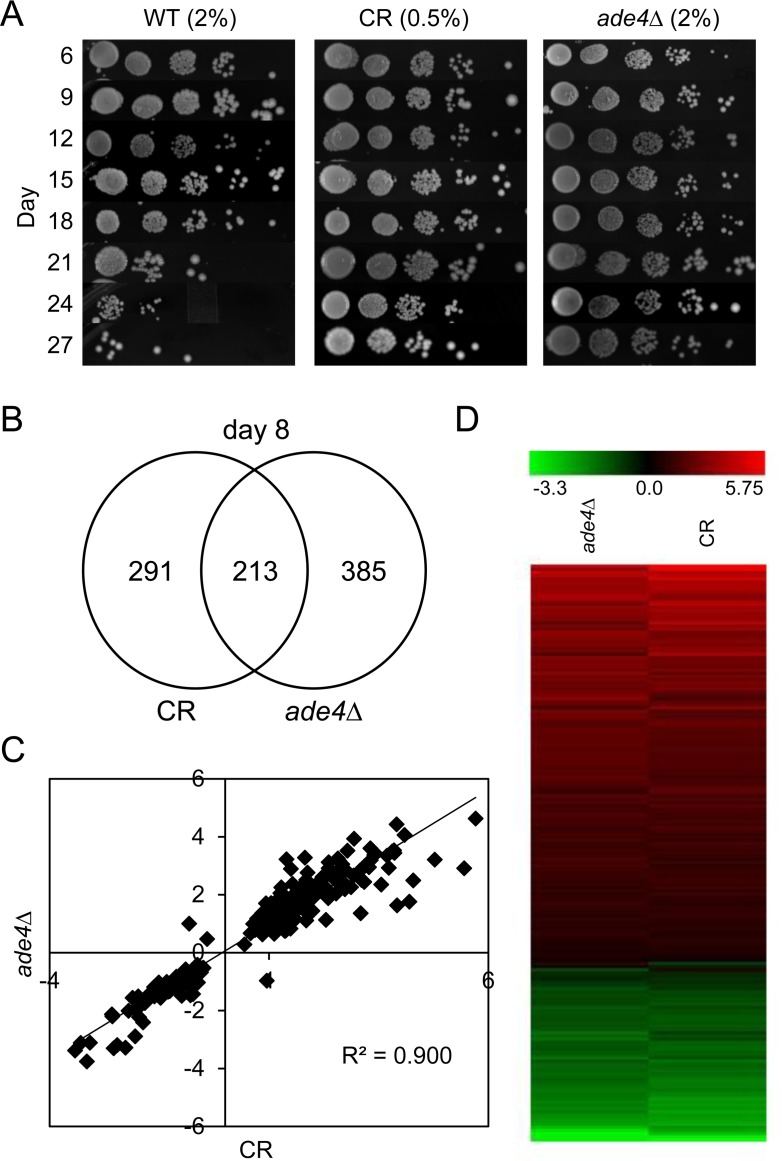
Overlapping effects of CR and *ade4∆* on chronological lifespan and gene expression (**A**) Semi-quantitative CLS assay showing the longevity effects for BY4741 (WT) under calorie restricted (CR) 0.5% glucose or non-restricted (NR) 2% glucose conditions, and with a non-restricted *ade4∆* mutant. (**B**) Venn diagram showing numbers of differentially regulated genes that overlap between CR and *ade4∆* conditions at day 8. (**C**) Scatter plot of gene expression fold-changes (log_2_) between CR and *ade4∆* conditions. (**D**) Heat map of gene expression fold-changes (log_2_) induced by CR or *ade4****∆*** relative to WT.

Our lab previously identified components of the *de novo* purine biosynthesis pathway as genetic factors with direct functional impact on CLS. Specifically, deletion of any *ADE* gene upstream of AICAR production was sufficient to significantly extend lifespan [9]. This extension, particularly for *ade4∆* (the first and rate-limiting step in the *de novo* pathway [19, 20]), was comparable to that observed for calorie restricted (0.5% glucose) cultures (Figure 1A). This finding, along with the observation that CR does not further extend the CLS of an *ade4∆* mutant, led us to hypothesize there was overlap between the mechanisms required for CLS extension induced by either CR or the *ade4∆* mutation [9].

To examine more specifically what molecular/cellular pathways could be involved in the overlap between CR and *ade4∆*, gene expression profiling analysis was performed on WT-NR, WT-CR, and *ade4∆* NR cultures grown to log or stationary phase (day 8). Day 8 was chosen to ensure the cells were in stationary phase and to potentially enrich for changes in transcription that occur during the aging process. Affymetrix Yeast Genome 2.0 GeneChips were used for the array hybridizations with duplicate biological RNA samples. Genes differentially regulated in each of the long-lived conditions compared to the non-restricted WT culture were identified using a 20% False Discovery Rate (FDR) cutoff for each combination. There were surprisingly no significant expression differences in the log phase cultures that passed this liberal FDR cut-off.

At day 8, however, the WT CR and *ade4∆* NR conditions were found to induce significant changes in the expression of 213 (p-value = 2.5e-90; 4-fold enrichment over random) common genes when compared to the WT NR cultures (Figure 1B, and Supplementary Table S1). Scatter plots (Figure 1C, R^2^=0.9002) and heat maps (Figure 1D) of the differential expression levels (log_2_) revealed that the relative changes in expression for the shared genes were also highly concordant. Given the large number of changes at day 8 for this dataset, we focused on this time point throughout the rest of the study.

### Effects of cell extrinsic factors on CLS and global gene expression

**Figure 2:**
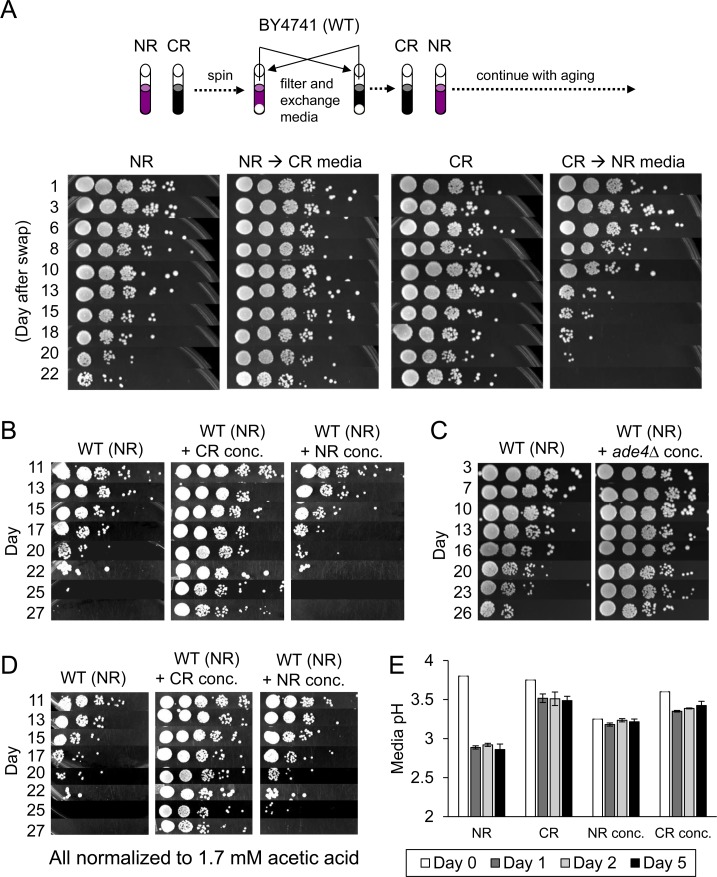
Extracellular effects of expired media on CLS (**A**) Schematic diagram depicting media exchange between centrifuged NR and CR cultures at day 5, followed by continued CLS assays over a 3-week period. (**B**) CLS assay showing effect of CR or NR concentrates when added to non-restricted BY4741 (WT) cultures. (**C**) CLS assay showing effect of expired media concentrate derived from a non-restricted *ade4∆* culture. (**D**) Same as panel B except each culture was normalized to 1.7 mM acetic acid at the time of inoculation. (**E**) Media pH measurements were taken at the time of inoculation (day 0), or days 1, 2, and 5 after inoculation. BY4741 was grown under NR and CR conditions or the cultures were treated with NR or CR concentrates.

CR and *ade4∆* conditions are known to have cell extrinsic effects on CLS, as demonstrated by previous media swapping experiments [9, 11]. In these studies, when the expired media from a non-restricted WT culture was replaced with the expired media from either a WT CR culture or an *ade4∆* NR culture, CLS was dramatically increased. An example of this is shown for a CR media swap in Figure 2A. The reciprocal result was observed when cells grown in CR media were transferred into expired NR media (Figure 2A, right panel). Since expired media from CR or *ade4∆* cultures contains less acetic acid, a simple interpretation was that swapping out acetic acid-laden media for acetic acid-depleted media was beneficial for CLS due to reduced toxicity [9, 11]. However, it was also possible that media from the CR or *ade4∆* cultures contained one or more secreted factors that extend CLS. To test this idea, we collected 50 ml of expired media from 5-day old WT CR or NR cultures, and then concentrated the volumes to 1 ml. These concentrates were then added to fresh WT NR cultures (10 ml total volume) and CLS was tracked. As shown in Figure 2B, the CR concentrate dramatically extended CLS, strongly suggesting there was something present in the CR concentrate that extends CLS, and was absent from the NR concentrate. Similarly, concentrate from the non-restricted *ade4∆* mutant also extended CLS when added to WT BY4741 growing in SC + 2% glucose (Figure 2C), further supporting the existence of extrinsic factors in the expired media.

We noticed there was residual acetic acid remaining in the NR concentrate (measured as 1.7 mM) that was absent in the CR concentrate and could potentially mask CLS extension by the NR concentrate. Along these same lines, it was also possible that the concentrated compounds in the CR concentrate could be buffering pH of the media and mitigating the negative lifespan effects of acid accumulation by WT yeast grown under NR conditions. To address the first possibility, we normalized the acetic acid to 1.7 mM for both the CR concentrate and NR concentrate and repeated the CLS assays. The CR concentrate again extended CLS and the NR concentrate did not (Figure 2C). To assess the buffering capacity of the NR and CR concentrates, the media pH of each culture was measured over the first 5 days of cell growth and stationary phase (Figure 2E). Although addition of the NR concentrate decreased the starting pH relative to the other cultures, by the first 24 hours there were no significant differences in media acidity between cultures treated with either the CR or NR concentrate, making pH buffering an unlikely cause for the effect on longevity. Experiments to identify positive CLS factors from these concentrates are ongoing, but for the purpose of the current study we have used the CR concentrate as a cell extrinsic means of extending CLS for microarray analysis and biomarker identification (see below).

**Figure 3:**
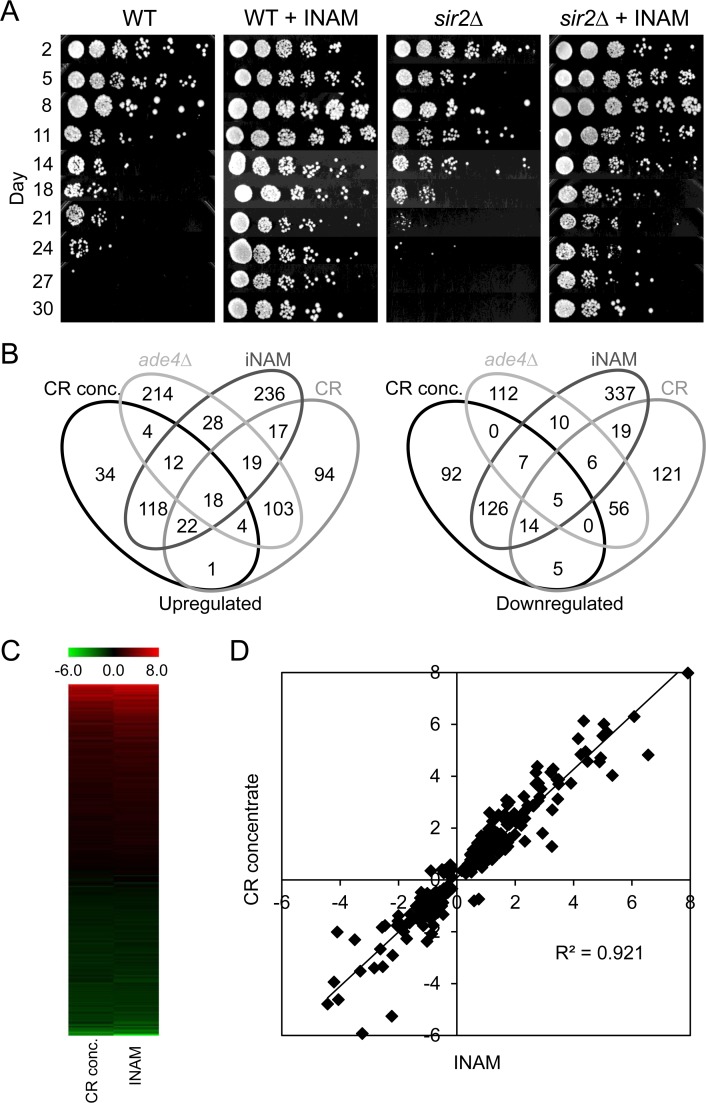
Overlapping effects of isonicotinamide (INAM) and expired CR media concentrate on CLS and gene expression (**A**) Isonicotinamide (25 mM), extends CLS of WT yeast through a *SIR2* independent mechanism. (**B**) Venn diagrams showing the number of genes found to be commonly up‐ or downregulated among multiple longlived conditions – CR conc., *ade4∆*, INAM, and CR– when compared to WT NR day 8 stationary cell populations. (**C**) Heat map of gene expression fold‐changes (log_2_) induced by INAM or the CR concentrate relative to WT. (**D**) Scatter plot of gene expression fold‐changes (log_2_) between day 8 cultures supplemented with the CR concentrate vs. INAM.

For the purpose of biomarker identification, we were interested in adding a 4^th^ long-lived growth condition for gene expression profiling that was unrelated to the CR concentrate. Isonicotinamide (INAM) is an isomer of nicotinamide that has been reported to enhance the NAD^+^-dependent histone deacetylase activity of sirtuins [21]. Although initially interested in its ability to promote Sir2 activity [22], we have also discovered the same concentration of INAM (25 mM) that enhances Sir2 activity *in vivo* also dramatically extends CLS, though independently of Sir2 or the other yeast sirtuins (Figure 3A and data not shown). Affymetrix expression profiles for the INAM and the CR concentrate were then generated from day 8 cultures for comparison with the CR and *ade4∆* datasets.

### Two distinct mechanisms of CLS extension revealed by expression profiles

Comparing expression profiles from the 4 long-lived populations (CR, *ade4∆*, INAM, and CR concentrate) relative to WT NR cultures revealed a wide diversity in the overlaps between each dataset, for both upregulated and downregulated genes (Figure 3B). This result was fully consistent with the disparate growth conditions used to extend CLS. Even with the 4-way comparison, the CR and *ade4∆* datasets still showed the most overlap with each other (213 genes). The CR concentrate and INAM datasets were also highly overlapping with each other, showing 336 combined genes whose differential expression levels were highly correlated as shown in a heat map (Figure 3C) and scatter plot (Figure 3D, R^2^=0.9234). These overlapping genes are listed in Supplemental Table S2. Given the relatively fewer genes shared across the other combinations, we hypothesized that INAM and the CR concentrate may extend CLS through a primary mechanism distinct from the effects of CR or the *ade4∆* mutation. In support of this idea, the top 10 GO terms for the upregulated and downregulated CR/*ade4∆* genes (Supplemental Table S3) were different from GO terms for the upregulated and downregulated INAM/CR concentrate genes (Supplemental Table S4).

The most prevalent GO terms for the CR and *ade4∆* samples indicated up-regulation of cellular processes related to the metabolism of organic acids such as acetic acid (Supplemental Table S3). By processing these metabolites, presumably in a manner that is energetically beneficial, CR and *ade4∆* also prevent the accumulation of extracellular organic acids, as has been previously demonstrated [9, 11]. On the other hand, GO terms for the INAM and CR concentrate samples indicated an up-regulation of cell wall structural components (Supplemental Table S4), a general feature associated with exposure to acetic acid [23, 24]. The GO term emphasis on cell wall organization implies that yeast in these cultures are most likely producing the same organic acids as WT cultures but are also restructuring their cell walls to better cope with an increasingly toxic environment. Varied downregulation GO terms for CR and *ade4∆* were not as informative, but included enrichment for transcription. For INAM and CR concentrate, the downregulation GO terms were enriched for vesicle and membrane fusion.

**Figure 4:**
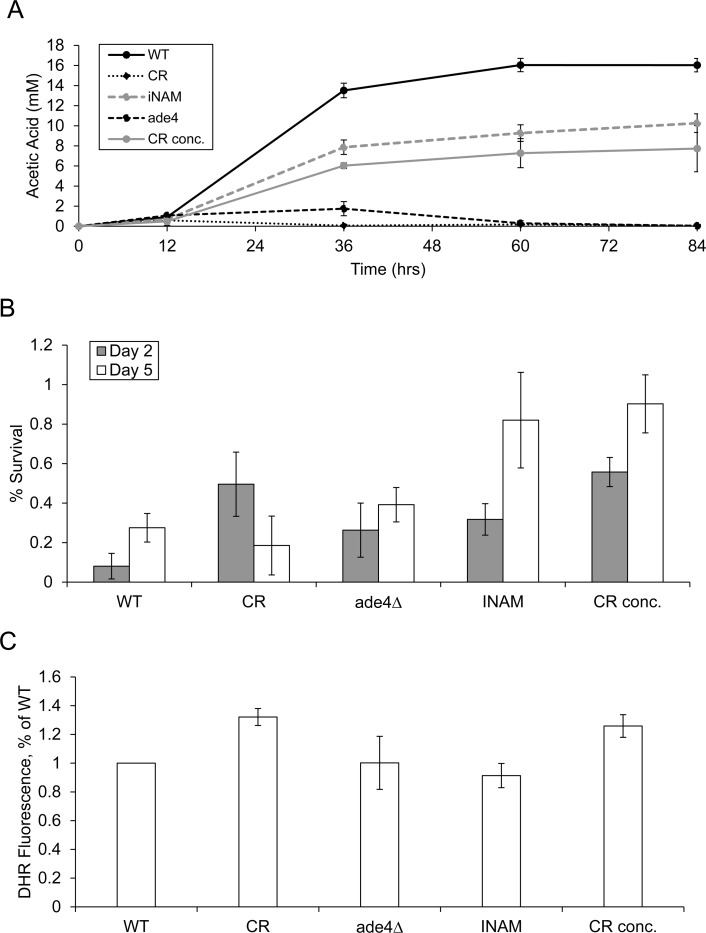
Effects of CLS-extending growth conditions on extracellular acetic acid accumulation and resistance to exogenous acetic acid (**A**) Accumulation of acetic acid in the media of CR, *ade4∆*, CR. conc. and INAM cultures was measured over the first 84 hours of the aging assays. (**B**) Cell viability was measured as colony forming units (CFU) following exposure to 300 mM acetic acid for 200 minutes. Exposures were performed at day 2 and day 5 of the aging assays. The fractions of viable cells in the treated cultures relative to untreated are plotted. (**C**) Assessment of ROS generation by long-lived cells using dihydrorhodamine (DHR 123) staining after 48 hr growth. Fluorescence intensity of the WT NR cells was normalized to 1.0.

Based on the clear distinction in GO terms, we next measured acetic acid levels in the growth medium for each of the long-lived culture conditions across an 84-hour time course. The CR concentrate and INAM cultures accumulated significant concentrations of acetic acid, albeit to a lesser degree than WT (Figure 4A). In contrast, the CR and *ade4∆* cultures accumulated very little acetic acid, even at the longer time points (Figure 4A), which was consistent with the up-regulation in organic acid metabolic processes and efficient consumption of acetic acid during the diauxic shift. The elevated acetic acid in INAM and CR concentrate cultures would theoretically require cells to become more resistant to the toxic environment in order to be long-lived, and thus the up-regulation of cell wall biosynthesis processes seems logical as functionally relevant GO terms. To model the extent that these differences in gene expression and resulting changes in metabolism could have on cell survival within each of the long-lived populations, we challenged each culture for 200 minutes with a toxic concentration of acetic acid (300 mM) at days 2 and 5 of the aging assay, and then measured the effects on cell viability (Figure 4B). As predicted from the array analysis and cell wall biosynthesis GO terms, INAM and CR-concentrate treated cells were best able to handle the acidic stress by day 5, maintaining significantly higher viability than WT, CR, and *ade4∆* cultures.

Interestingly, all four long-lived populations exhibited slightly higher acetic acid tolerance at day 2 than WT before diverging into two distinct phenotypic subgroups, leaving open the possibility that there may still be some common cellular events occurring during early stationary phase responsible for all four increases in lifespan. To investigate this further, we evaluated the long-lived populations for other phenotypic features associated with increased longevity. Specifically, we checked ROS levels at early stationary phase (48 hours) as an indication of changes in mitochondrial function known to effect lifespan (Figure 4C). We could not, however, discern a common pattern among all of the conditions, with only CR and the CR concentrate exhibiting increased ROS levels. It is, therefore, unlikely that a common change in mitochondria function is responsible for the CLS increase observed in all four populations, though it is intriguing that both CR-associated conditions show the increase in ROS.

### Comparative gene expression profiling of all 4 long-lived populations yields potential biomarkers of extended CLS

**Figure 5:**
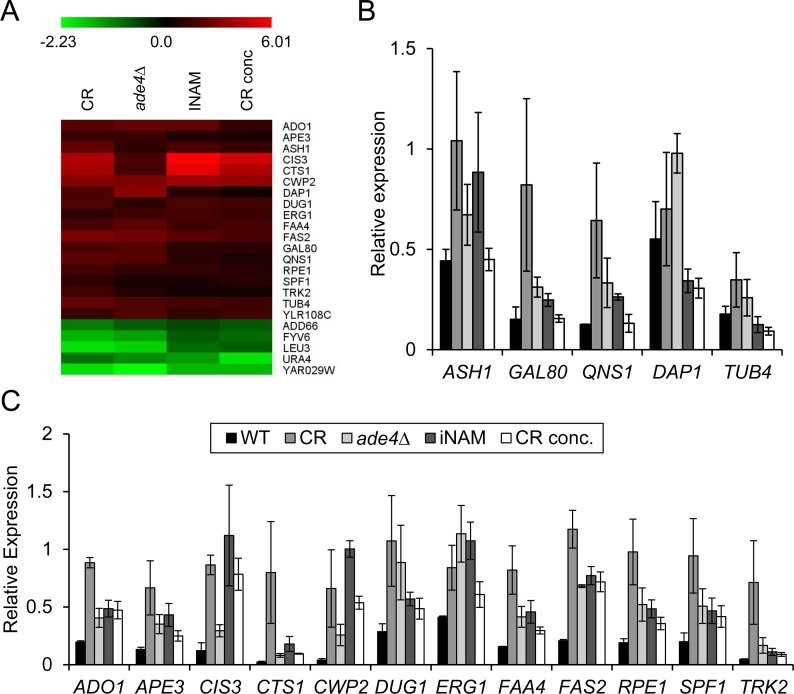
Comparative microarray analysis reveals potential biomarkers of CLS (**A**) Heat map of log_2_ fold-expression changes of 23 genes that are differentially regulated in all the long-lived condition when compared to WT non-restricted cultures. (**B**) qRT-PCR of expression changes for 5 genes from the upregulated class that could not be confirmed as statistically changed in all 4 conditions. (**C**) qRT-PCR of expression changes for the 12 genes shown to be significantly upregulated at day 8 (p<0.05). All qRT-PCR data was normalized to *ACT1* transcript levels.

**Figure 6:**
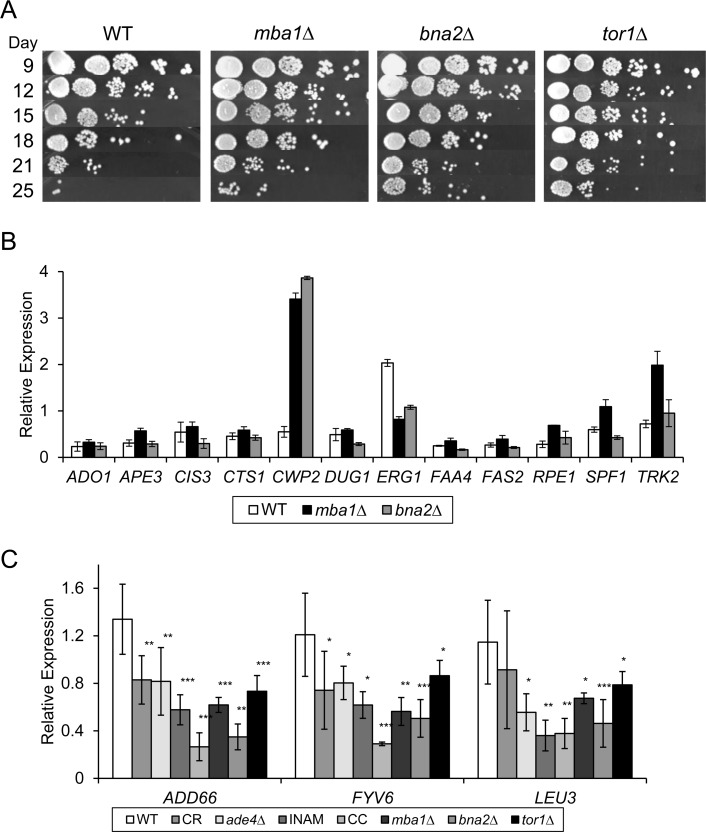
Testing efficacy of putative gene expression CLS extension biomarkers (**A**) Single gene deletion mutants (*mba1∆*, *bna2∆*, and *tor1∆*) extend CLS and provide test cultures to evaluate the predictive value of the potential biomarkers identified through the microarray analysis. (**B**) Quantitative RT-PCR analysis of putative upregulated gene biomarkers in the *mba1∆* and *bna2∆* mutants. (**C**) Quantitative mRNA levels of *ADD66*, *LEU3*, and *FYV6* are downregulated in all tested long-lived populations relative to WT at day 8.

Hidden among the diversity of gene expression changes between the 4 different lifespan extending conditions, were 18 commonly upregulated and 5 downregulated genes (Figure 3B and 5A). By qRT-PCR with a p-value cut-off of 0.05, we could confirm 12 of the 18 upregulated genes in all four conditions (Figure 5B and C), and 3 of the 5 downregulated genes (Figure 6C). For one of the downregulated genes, *YAR029W*, its mRNA was below the level of detection for RT-PCR confirmation (data not shown). To test the predictive value of these 15 confirmed genes as biomarkers for CLS extension, we used qRT-PCR to measure their expression from two additional long-lived deletion mutants, *mba1∆* and *bna2∆*, that were randomly selected from a previous genome-wide screen for longevity genes [9]. Although both mutants consistently showed extended CLS (Figure 6A), we could only show significant expression changes for 1 of the original 12 upregulated genes, *CWP2*, in both strains at day 8 (Figure 6B). Most of the upregulated genes other than *CWP2* are therefore not effective biomarkers for CLS extension beyond the 4 original long-lived conditions used for the expression profiling. However, CLS extension for the *mba1∆* and *bna2∆* mutants was not nearly as extensive compared to CR, *ade4∆*, INAM, or the CR-concentrate, making it possible that most of these commonly upregulated genes only hold predictive value in cases of more extreme CLS extension.

Success at validating biomarkers improved with the 3 confirmed downregulated genes - *LEU3*, *ADD66*, and *FYV6*. Similar to the initial 4 long-lived conditions, expression of all three genes was downregulated in the *mba1∆* and *bna2∆* mutants on day 8 (Figure 6C). For additional validation we measured expression of the downregulated genes in a *tor1∆* mutant that was well known to extend CLS [10]. Expression of *LEU3*, *ADD66*, and *FYV6* was also reduced in the *tor1∆* mutant (Figure 6C), making it possible that down-regulation of these 3 genes is a common feature of chronologically long-lived yeast strains, and suggesting they could be used as biomarkers for future screening purposes.

**Figure 7:**
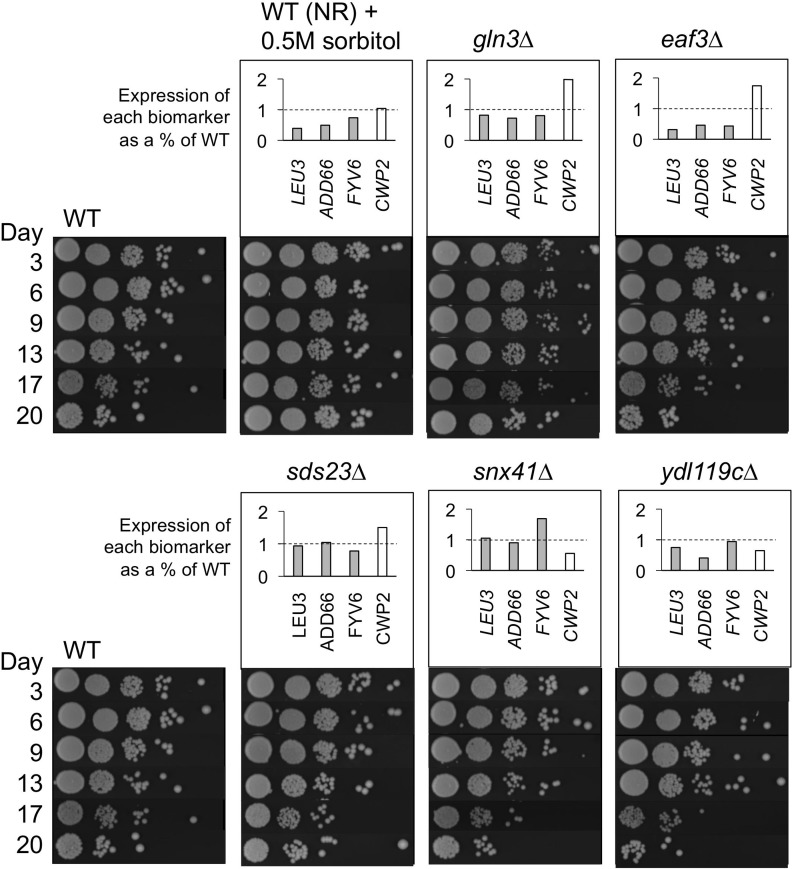
Randomized blinded experiment to test utility of CLS extension biomarkers Cultures for each strain or growth condition were randomized and analyzed for CLS or expression of *LEU3*, *ADD66*, *FYV6*, and *CWP2* relative to the non-restricted WT control strain. Expression of the WT NR strain was normalized to 1.0. *LEU3, ADD6*, and *FYV6* expression values below 1 (dashed line) and *CWP2* expression above 1 (dashed line) are predictive of long CLS. The WT + sorbitol culture and the *gln3∆* mutant were chosen as long-lived conditions. The *eaf3∆*, *sds23∆*, *snx41∆*, and *ydl119C∆* mutants were randomly chosen from the KO collection.

To this end, we designed a small blinded experiment to test the predictive power of the four CLS biomarkers (*LEU3*, *ADD66*, *FYV6*, and *CWP2*). Two known long-lived conditions, 0.5M sorbitol treatment and a *gln3∆* mutant, were chosen as test cases [10, 25], along with WT BY4741 and 4 randomly selected deletion mutants from the yeast KO collection. Each sample was blindly assigned a random numerical value by another lab member, and then CLS and relative mRNA biomarker expression were tested in triplicate (Figure 7). The average expression level of each biomarker was normalized to the corresponding average of the WT. Those samples with normalized expression values <1 for the three downregulated biomarkers and a value >1 for the upregulated biomarker *CWP2* were predicted to be long-lived (sorbitol, *gln3∆*, and *eaf3∆*). When compared to the CLS data, we had successfully predicted the lifespan for 5 out of 6 strains, with the *eaf3∆* mutant called as a false positive for extended CLS.

### Comparative gene expression profiling of long-lived populations yields genes with a functional impact on CLS

**Figure 8:**
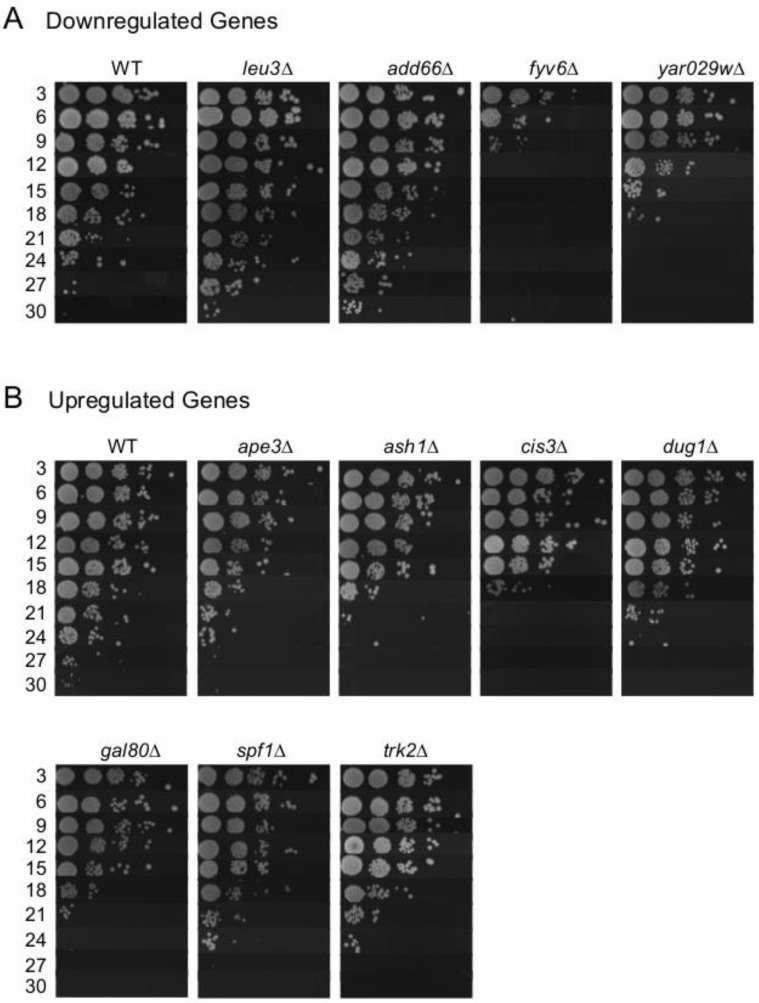
Effects of deleting putative expression biomarker genes on CLS (**A**) Downregulated gene deletions that alter CLS compared to WT. (**B**) Upregulated gene deletions that alter CLS compared to WT.

While only 4 of the commonly regulated genes were found to be effective biomarkers of CLS extension, we hypothesized that the overall list was enriched for genes that functionally impact aging in the BY4741 background. To test this idea, we obtained deletion mutants from the yeast knockout collection for 21 of the 23 original shared genes and determined their relative CLS (Figure 8). Of the mutants not shown, *fas2∆*, *qns1∆*, and *erg1∆* could not be tested because the genes are essential, and the others had no effect on CLS d to WT. The rationale to also test deletion mutants for the unconfirmed genes was because *YAR029W* expression was below detection limits, and *ASH1*, *GAL80*, *QNS1* were either trending or significantly upregulated in the CR, *ade4∆*, and INAM cultures (Figure 5B), suggesting they could still be functionally relevant. We initially predicted that deleting a gene that is downregulated during aging would be beneficial to stationary phase survival and result in CLS extension. However, while all four of the downregulated deletion mutants functionally impacted CLS, only *leu3*∆ and *add66∆* were long-lived compared to WT (Figure 8A). Extended CLS of the *leu3∆* mutant was reported previously [26, 27]. Add66 is involved in proteasome assembly and the function of *YAR029W* remains unknown. Fyv6 has a stationary phase specific role in double stranded break repair [28], which may explain poor survival of the *fyv6∆* mutant (Figure 8A).

The upregulated genes we tested for functional relevance had less variation in their CLS effect. Of the 12 tested deletion mutants, 8 had a measurable impact on CLS (Figure 8B). Of these 8 mutants, 7 had reduced CLS relative to WT, which was anticipated if up-regulation of these genes was beneficial to stationary phase survival. However, none of the CLS reductions were severe compared to WT (all mutants persisted until day 18 at least), making it unlikely that any of these factors are individually major contributors to stationary phase survival. Instead, the data are consistent with the idea that yeast chronological lifespan is defined by a complex combination of numerous factors, and is a highly multigenic process regulated at the transcriptional level.

### Mutations in the adenosine salvage pathway extend CLS similarly to those in the adenine salvage pathway

**Figure 9:**
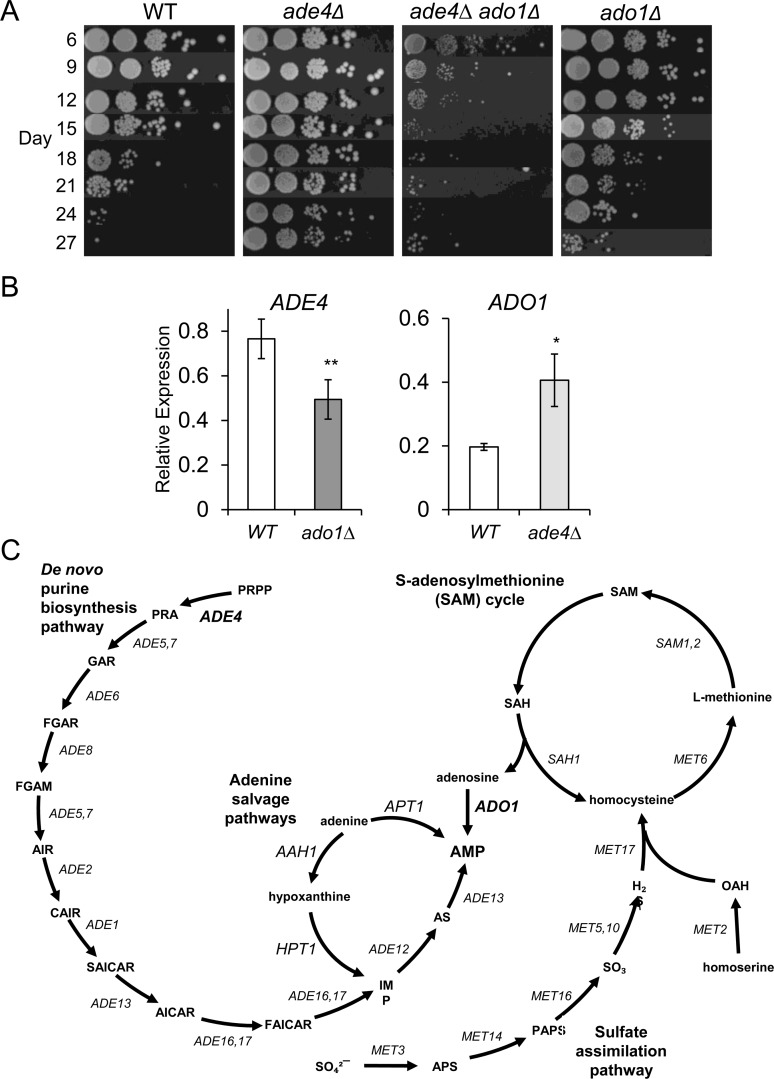
Genetic interactions between *ADE4* and *ADO1* (**A**) CLS assays with WT, *ade4∆*, *ado1∆*, and *ade4∆ ado1∆* mutant strains. (**B**) Quantitative RT-PCR showing relative downregulation of *ADE4* in an *ado1∆* mutant and the upregulation of *ADO1* in an *ade4∆* mutant. All expression levels were first normalized to *ACT1* and then compared to WT. (**C**) AMP convergence of the *de novo* purine biosynthesis pathway with the various purine salvage pathways. Ado1 predominantly converts adenosine derived from the SAM cycle.

The only shared upregulated gene to cause CLS extension when deleted was *ADO1* (Figure 9A), which encodes an adenosine kinase that salvages adenosine derived from the methyl cycle by converting it to AMP [29]. Ado1 also converts 5-amino-4-imidazole-carboxamide ribonucleoside (AICAR) into the active phosphorylated form [30]. While the extended CLS for this mutant was weaker than deletions of *de novo* purine biosynthesis genes such as *ade4∆* (Figure 9A), it was similar to the effects of deleting *APT1* or *AAH1* [9], both of which are involved in salvaging adenine to produce AMP (see adenine salvage pathway in Figure 9C). We had earlier proposed that the modest lifespan extension caused by mutants such as *apt1∆* or *aah1∆* was possibly due to secondary effects on expression of the *de novo* purine synthesis pathway [9]. To test this idea for the *ado1∆* mutant, we analyzed the effect of this mutation on *ADE4* expression and found it to be reduced at day 8 (Figure 9B), consistent with such a model. On the other hand, up-regulation of *ADO1* in the *ade4∆* mutant (Figure 5C and 9B) suggested that adenosine salvage from the methyl cycle was important in compensating for loss of *de novo* purine biosynthesis. Indeed, deleting *ADO1* completely blocked the *ade4∆*-induced CLS extension, with the double mutant having a CLS even shorter than WT (Figure 9A). Eliminating two pathways to AMP synthesis was apparently too detrimental for proper stationary phase survival.

## DISCUSSION

The diversity of lifespan-extending conditions used for microarray analysis in this study resulted in a small number of genes with similar expression level changes. We were initially expecting to observe more overlap, but the limited number was consistent with mechanistic contributions from numerous pathways, as might be predicted for a phenomenon as complex as aging. *CWP2* was upregulated in all the tested conditions, while *LEU3*, *ADD66*, and *FYV6* were downregulated. It is important to emphasize that the up- and downregulation in these comparisons are relative to a WT strain grown in standard SC media with 2% glucose (NR) for eight days. Numerous genes are repressed at some level during stationary phase [31, 32], and *CWP2* for example, is simply not repressed as much in the long-lived conditions (thus upregulated compared to the WT condition), while *LEU3*, *ADD66*, and *FYV6* are repressed even more than normal (thus downregulated). When taken together as a group, these four genes are potentially predictive of whether a particular strain or SC medium manipulation will result in CLS extension, which was demonstrated with a blind experiment in Figure 7. These biomarkers were identified and confirmed with the yeast KO collection strain background BY4741, and should be effective in screening with this commonly used resource. Future analysis will be needed to determine the extent of their usefulness in other strain backgrounds.

At the single gene level, *LEU3* is intriguing as a potential biomarker of CLS extension because it has been previously implicated in longevity control [26, 33]. *LEU3* encodes a zinc-knuckle transcription factor that regulates genes involved in branched chain amino acid biosynthesis, including *LEU2*, which is commonly mutated in lab strains. The CLS of a *leu2∆* auxotrophic strain is shorter than a *LEU2* prototrophic strain [26], and supplementing minimal medium with excess branched chain amino acids (leucine, isoleucine, valine) is sufficient to extend CLS [26]. Taken together this implies that leucine availability and/or branched chain amino acid homeostasis are important for maintaining long-term viability. Similarly, branched chain amino acids have been implicated in longevity of higher eukaryotes [34, 35]. *LEU3* itself is a target for transcriptional regulation by the general amino acid control network mediated by Gcn4 [36]. The extended CLS induced by branched chain amino acid supplementation is also suppressed by *GCN4* over-expression [26]. The BY4741 strain used for experiments in the current study has a *leu2∆0* mutation, making it a leucine auxotroph. The identification of *LEU3* as a gene expression biomarker of general CLS extension is therefore in the context of a *leu2∆* auxotroph that is supplemented with leucine in the growth medium to permit cell growth. It is possible that down-regulation or deletion of *LEU3* is necessary for BY4741 to efficiently utilize exogenous leucine or maintain proper homeostasis when endogenous biosynthesis is blocked by the *leu2∆* mutation.

Identifying *LEU3* as one of the longevity biomarkers is also intriguing because leucine metabolism has been linked to ketone body production in mammals and acetic acid production in yeast, though the mechanism in yeast remains mysterious [16]. Like ketone bodies, acetic acid in the form of acetate is converted to acetyl CoA, which then enters the TCA cycle in order to contribute to energy production. Low media pH drives acetate into the cell by passive diffusion across the plasma membrane, and ultimately accumulates in the mitochondria, which can inhibit metabolic functions [37]. Acetic acid in the mitochondria is “detoxified” by conversion to acetyl CoA via the CoA transferase Ach1 [37], and to a lesser extent by the acetyl CoA synthetase Acs1. Deletion of *ACH1*, but not *ACS1*, dramatically shortens CLS [16]. Furthermore, deletion mutants of *SCH9* or *TOR1*, both of which extend CLS [10, 38], show enhanced Ach1-mediated acetic acid consumption that is also dependent on an active electron transport chain. This acetic acid consumption is accompanied by the accumulation of trehalose [16], a stress-resistance carbon source that has been implicated in CLS extension via CR [39]. CR has been shown to prime yeast cells for more efficient oxidative metabolism by promoting ethanol catabolism [40], which is necessary to reach a critical mitochondrial respiratory threshold for lifespan CLS extension [41]. Our results showing CR-mediated acetic acid consumption are consistent with these findings and suggest that CR and the *ade4∆* deletion also trigger a metabolic program related to ketone body catabolism, as emphasized by the enrichment for carboxylic acid and cellular ketone related GO terms (Supplemental Table S3).

Technical differences in cell culturing that affect aeration levels and media evaporation rates appear to impact CLS in synthetic medium [11, 16]. In our hands, the extracellular acetic acid concentration using 15 ml glass culture tubes on a roller drum reaches a maximum of 10 to 16 mM with BY4741 growing in SC 2% glucose medium. While this concentration does not affect cell viability over a short-term exposure, it could have detrimental effects on viability long term under low pH conditions, such as stationary phase cultures, or especially specific hypersensitive mutants. Our results imply that acetic acid consumption and protection from toxicity both likely contribute to CLS during CR in SC medium, though they are not necessarily mutually exclusive. While CR and the *ade4∆* mutation both promoted acetic acid consumption, INAM and the CR concentrate extended CLS without dramatically enhancing acetic acid consumption, though there was still a significant small reduction in overall accumulation in the media (Figure 4A). Despite the strong link in our study between CLS and acetic acid, there are clearly other mechanisms of CLS extension that do not involve acetic acid, including direct increases in mitochondrial respiration and adaptive ROS signaling [41, 42], which is consistent with the moderately increased ROS levels we observed with CR or the CR concentrate (Figure 4C).

Consumption of acetic acid/acetate, regardless of whether it occurs in the mitochondria, cytoplasm, or nucleus, results in production of acetyl CoA. Interestingly, variations in the intracellular acetyl-CoA concentration were recently shown to impact CLS, and it is also possible that the accumulation of acetic acid may be indicative of a lack of acetyl-CoA production [43-45]. These findings may hint at an underlying mechanism for CR and *ade4∆*-induced chronological longevity. Specifically, it has been found that acetyl-CoA levels in yeast increase as a result of increased Snf1 (AMPK) activity and the mitochondria retrograde response pathway [44]. Snf1 is a master regulator of many cellular pathways and is activated under low glucose conditions [4], consistent with the idea that Snf1 would be important for CLS under CR conditions. Heterozygous mutations of *de novo* purine biosynthesis genes in *Drosophila* have been shown to surprisingly activate AMPK and extend adult lifespan [46]. An adenosine kinase heterozygous mutant was also shown to extend lifespan in the same study, reminiscent of the extended CLS we observe with the *ado1∆* mutant in Figure 9. It is therefore possible that *de novo* purine synthesis pathway mutants such as *ade4∆* also trigger Snf1-dependent CLS extension in yeast. Similarly, loss of *ADO1* likely alters the relative adenine nucleotide levels or ratios in a manner sufficient for lifespan extension.

The gene expression biomarkers identified using this microarray comparison approach are clearly effective using SC growth medium and the BY4741 strain background. However, there are numerous instances of strain background and media composition significantly altering CLS. For example, simply buffering the pH of SC medium to 6.0 is sufficient to extend lifespan [11]. Additionally, the *met15∆0* mutation in BY4741 was recently shown to extend CLS by mimicking methionine restriction [47, 48], another dietary regimen that extends lifespan from yeast to mammals [49, 50]. All the experiments in this study are therefore likely in the context of a mild methionine restriction-like background. Ado1 salvages adenosine from the S-adenosylmethionine cycle (Figure 9C), raising the possibility of a link with methionine restriction, and perhaps effects on hydrogen sulfide accumulation, which was recently shown to be important in mediating methionine restriction and CR effects on lifespan [51]. Another example of media affecting lifespan is a recent study showing that in rich YPD medium, mutations in the *de novo* purine biosynthesis pathway actually shorten CLS [27]. We have also shown that supplementing SC media with excess adenine reverts the long-lived *ade4∆* phenotype back to normal CLS [9]. The extreme sensitivity of yeast CLS to growth conditions and strain background may account for most of the differences in results and models between various studies. There is potentially a core of genetic anti-aging and pro-aging factors/processes such as autophagy and mitochondrial respiration that are critical regardless of the growth condition [8, 9, 27, 52]. Others are more specific or uncovered within a particular system, but still potentially informative about general mechanisms of aging.

## METHODS

### Yeast strains and media

The wild type strain used in this study was BY4741 (*MAT*a *his3∆1 leu2∆0 met15∆0 ura3∆0*). Strains containing gene deletions were obtained yeast knockout collection [53], or generated by PCR-mediated gene replacement using the G418 resistance marker *KANMX4* from pRS400 [54]. Synthetic complete (SC) media was used for all experiments, with glucose added to a final concentration of 2.0% (NR; non restricted) or 0.5% (CR; calorie restricted) [9, 55]. For isonicotinamide (INAM) cultures, a 1 M INAM stock was used to bring the final concentration to 25 mM INAM in the SC media. All cultures were grown at 30°C.

### Chronological lifespan assays

Semi-quantitative CLS assays were performed as described previously [25]. Briefly, overnight 10 ml cultures were started from single colonies and grown at 30°C in 15 ml glass culture tubes with loose fitting metal caps on a New Brunswick Scientific roller drum. 100 μl of the overnight cultures was used to inoculate fresh 10 ml cultures in the indicated SC medium. For each time point, 20 μl aliquots were removed and serially diluted 1:10 with sterile H_2_O in a 96-well plate. Then 2.5 μl of each dilution was spotted onto YPD plates and colonies allowed to grow for 2 days to measure cell viability. Digital photos were then taken of each plate. At the end of each experiment, images of the serial dilutions on YPD plates for each sample and time point were cropped and compiled into a single image of the time course.

### Media swap

BY4741 was grown in 10 mL SC cultures with either 2.0% (NR) or 0.5% (CR) glucose at 30°C for 5 days. On day 5, cultures were centrifuged at 2,500 RPM in an Eppendorf 5810-R table-top centrifuge with a swinging buck rotor. The supernatants were collected and passed through a 0.2 micron filter to remove residual cells. The cell pellets were then resuspended in the opposite sterilized culture supernatant and the swapped cultures allowed to age at 30°C in the roller drum. Semi-quantitative CLS assays were then carried out at the indicated time points.

### Expired media concentrate

BY4741 was grown in 50 mL SC cultures (with either NR or CR levels of glucose) at 30°C for 5 days. The cultures were centrifuged at 2,500 RPM in an Eppendorf 5810-R table-top centrifuge with a swinging buck rotor. The supernatants were collected and condensed from 50 mL down to 1 mL using a Büchi Rotavapor-R apparatus, then sterilized by passing through a 0.2 micron filter. For each experimental culture, 1 mL of media concentrate (derived from either CR or NR cultures) was added to 9 mL of non-restricted SC media. As a control, 1 ml of sterile H_2_O was added instead. The cultures were then inoculated and CLS measured. For the *ade4∆* media concentrate, the *ade4∆* mutant (SY403) from the yeast knockout collection was grown in 50 mL of SC (NR), and then the expired media concentrated in the same manner.

### Microarray analysis

Total RNA was harvested from duplicate 10 mL BY4741 NR, CR, CR conc., INAM, and *ade4∆* (SC, NR) cultures on day 8 using the acid phenol method [56]. The gene expression profile of each sample (4 μg RNA) was then determined by using Affymetrix Yeast Genome 2.0 GeneChip arrays as previously described [57]. Raw data from the CEL files were quantile normalized and expression values estimated using GCRMA in Bioconductor [58]. Lists of differentially expressed genes for all pair-wise comparisons (i.e. long-lived condition versus normal control) were derived using Limma [59], which applies a modified t-test, correcting for multiple hypothesis testing by applying False Discovery Rate (FDR) correction to the p-values [58] and applying a 20% FDR cutoff. Hierarchical cluster heat maps of differential expression levels (log_2_) between the long-lived conditions versus normal control samples of genes that passed the 20% FDR cutoff (y-axis) vs. long-lived condition (x-axis) were generated using TM4 MultiExperiment Viewer. The Affymetrix array data sets have been deposited in the NCBI Gene Expression Omnibus, and are accessible through GEO Series accession number GSE61341.

### qRT-PCR confirmation of microarray data

cDNA was synthesized from 1 μg total RNA using a High Capacity cDNA Reverse Transcriptase Kit (Applied Biosystems) per manufacturer's instructions, then quantitated by PCR reactions using SYBR Hi-ROX Mastermix (Bioline) and a StepOnePlus Real Time PCR System (Applied Biosystems). Samples were isolated from day 8 cultures. Transcript levels were normalized to levels of *ACT1* mRNA. Standard deviation for expression level of each transcript of interest was calculated for each condition.

### Randomized, blinded test of biomarker utility

We chose 2 independent means of extending CLS for this experiment. A long-lived *gln3∆* mutant was used and the WT BY4741 strain was also treated with 0.5M sorbitol to extend CLS by osmotic stress [25]. Four single gene deletion strains of unknown chronological lifespan were also randomly selected from the BY4741 knockout collection (*snx41∆*, *eaf3∆*, *ydl119c*∆, and *sds23∆*). Each test strain and the BY4741 control were grown in 2 sets of biological triplicates (10 mL SC 2% glucose media). Each triplicate set was assigned an independent, random numerical value by a third party. The CLS of one set of cultures was assessed using the semi-quantitative CLS assay, and the other was used for measuring the relative mRNA expression of each of the putative biomarkers *LEU3*, *ADD66*, *FYV6*, and *CWP2* using qRT-PCR (described above) on day 8. The average expression values for each set of unknown triplicate was then normalized to the known WT expression values. Those samples with normalized values <1 for the downregulated biomarkers (*LEU3*, *ADD66*, and *FYV6*) and >1 for the upregulated biomarker (*CWP2*) were predicted to correspond to the long-lived samples of the first set.

### Acetic acid measurements and treatments

To measure acetic acid in the growth media, 100 μL aliquots were taken at designated time points from each aging 10 mL culture. Cells were pelleted by centrifugation (2,500 rpm at 4°), and 50 μL of supernatant was removed and stored at −80°C. Acetic acid concentration for each sample was then later determined using the Acetic Acid Kit (Biopharm AG) per manufacturer's instructions. The acetic acid concentrations and standard deviations provided are an average of three biological replicates for each condition. To assess sensitivity to extracellular acetic acid, the cell viability of each aging culture was quantitated before and after treatment with 300 mM acetic acid for 200 minutes at 30°C. To quantitate viability, 20 μl aliquots were taken from each culture, diluted in water, and spread onto YPD (2% glucose) plates. The total number of colonies for each group was counted and the viability ratio of pre-acetic acid treatment to post-treatment was calculated. Acetic acid tolerance was assessed on both day 2 and day 5 of aging cultures for each condition. For each condition and time point, three biological replicates were tested.

### ROS measurements

Cell cultures were started at OD_600_ = 0.015 in SC medium. After 48 hours, cells were harvested and washed with incubation buffer (10 mM MES, 0.1 M KCl, 0.14 M NaCl, pH 6.5). Equal numbers of cells (1×10^7^) for each condition were resuspended in 1 mL of incubation buffer. Dihydrorhodamine 123 (1 mg/ml) was added to each reaction (1×10^7^cells/ml) to obtain a final concentration of 5 μM. Cells were incubated for 10 min at room temperature, and the fluorescence intensity was then measured using a Molecular Devices Spectramax M2 plate reader at excitation 504/emission 534 nm. For each condition, three biological replicates were tested and normalized to WT NR samples.

## SUPPLEMENTARY TABLES








