# Supplementary material for: Functional genomic analysis reveals overlapping and distinct features of chronologically long-lived yeast populations
Source: Aging (Albany NY). 2015 Mar 7;7(3):177–94. doi: 10.18632/aging.100729 (PMC4394729; doi:10.18632/aging.100729)
Supplement: Supplementary file 3 [file aging-07-0177-s003.pdf]

Supplemental Table S3. GO terms of genes differentially regulated by CR and *ade4*Δ

Upregulated terms

GOBPID	Pvalue	OddsRatio	ExpCount	Count	Size	Term
GO:0006519	0	8.615	7	41	318	cellular amino acid and derivative metabolic process
GO:0006082	0	7.17	9	45	419	organic acid metabolic process
GO:0044281	0	5.122	23	69	1026	small molecule metabolic process
GO:0019752	0	6.802	9	42	400	carboxylic acid metabolic process
GO:0043436	0	6.802	9	42	400	oxoacid metabolic process
GO:0044106	0	8.137	6	35	273	cellular amine metabolic process
GO:0042180	0	6.531	9	42	414	cellular ketone metabolic process
GO:0044283	0	6.475	9	42	417	small molecule biosynthetic process
GO:0006520	0	8.291	6	33	250	cellular amino acid metabolic process
GO:0009308	0	7.407	7	36	306	amine metabolic process

Downregulated terms

GOBPID	Pvalue	OddsRatio	ExpCount	Count	Size	Term
GO:0019219	0	3.722	8	22	767	Regulation of nucleobase, nucleoside, nucleotide and nucleic acid metabolic process
GO:0051171	0	3.711	8	22	769	Regulation of nitrogen compound metabolic process
GO:0045449	0	3.216	7	18	675	Regulation of transcription
GO:0031323	0	2.723	11	23	1056	Regulation of cellular metabolic process
GO:0080090	0	2.723	11	23	1056	Regulation of primary metabolic process
GO:0019222	0	2.607	12	24	1155	Regulation of metabolic process
GO:0006350	0	2.915	8	18	735	transcription
GO:0050794	0	2.466	15	27	1406	Regulation of cellular process
GO:0051301	0.001	3.522	4	11	349	Cell division
GO:0060255	0.001	2.49	11	21	1014	Regulation of macromolecule metaboic process
