# Supplementary material for: Functional genomic analysis reveals overlapping and distinct features of chronologically long-lived yeast populations
Source: Aging (Albany NY). 2015 Mar 7;7(3):177–94. doi: 10.18632/aging.100729 (PMC4394729; doi:10.18632/aging.100729)
Supplement: Supplementary file 4 [file aging-07-0177-s004.pdf]

Supplemental Table S4. GO terms of genes differentially regulated by INAM and CR concentrate

Upregulated terms[1]

GOBPID	Pvalue	OddsRatio	ExpCount	Count	Size	Term
GO:0071555	0	4.614	7	26	259	cell wall organization
GO:0007047	0	4.614	7	26	259	cellular cell wall organization
GO:0045229	0	4.614	7	26	259	external encapsulating structure organization
GO:0071554	0	4.236	8	27	291	cell wall organization or biogenesis
GO:0070882	0	4.067	8	26	289	cellular cell wall organization or biogenesis
GO:0006629	0	3.749	8	25	297	lipid metabolic process
GO:0031505	0	5.438	4	16	132	fungal-type cell wall organization
GO:0008610	0	4.503	4	17	166	lipid biosynthetic process
GO:0016126	0	10.862	1	8	36	sterol biosynthetic process
GO:0006694	0	10.862	1	8	36	steroid biosynthetic process

Downregulated terms[2]

GOBPID	Pvalue	OddsRatio	ExpCount	Count	Size	Term
GO:0048284	0	6.55	2	10	76	organelle fusion
GO:0006904	0	13.948	0	4	16	vesicle docking involved in exocytosis
GO:0006906	0.001	6.178	1	6	47	vesicle fusion
GO:0016071	0.001	2.586	7	16	286	mRNA metabolic process
GO:0048278	0.002	8.799	1	4	23	vesicle docking
GO:0022406	0.002	8.358	1	4	24	membrane docking
GO:0006944	0.003	4.055	2	7	80	cellular membrane fusion
GO:0061025	0.003	4.055	2	7	80	membrane fusion
GO:0051099	0.003	41.353	0	2	4	positive regulation of binding
GO:0006397	0.006	2.496	5	12	218	mRNA processing
